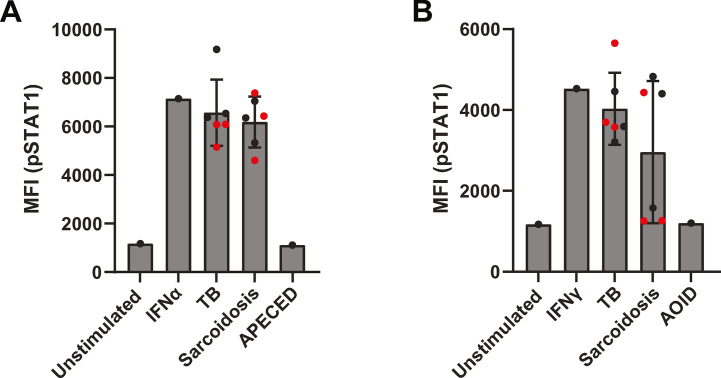# Increased serum interferon activity in sarcoidosis compared to that in tuberculosis: Implication for diagnosis?

**DOI:** 10.1016/j.heliyon.2024.e37103

**Published:** 2024-08-28

**Authors:** Benjamin Schrijver, Jens Göpfert, Rina La Distia Nora, Ikhwanuliman Putera, Nicole M.A.N. Nagtzaam, Marja A.W. Smits te Nijenhuis, Angelique L.C.T. van Rijswijk, Josianne C.E.M. ten Berge, Jan A.M. van Laar, P. Martin van Hagen, Willem A. Dik

**Affiliations:** a Laboratory Medical Immunology, Department of Immunology, Erasmus MC University Medical Center Rotterdam, the Netherlands; b Department of Applied Biomarkers and Immunoassays, NMI Natural and Medical Sciences Institute at the University of Tübingen, Reutlingen, Germany; c Department of Ophthalmology, Faculty of Medicine, Universitas Indonesia, Cipto Mangunkusumo Hospital, Jakarta, Indonesia; d Department of Internal Medicine, section Allergy & Clinical Immunology, Erasmus University Medical Center, Rotterdam, the Netherlands; e Department of Ophthalmology, Erasmus MC University Medical Center Rotterdam, the Netherlands

**Keywords:** Tuberculosis, Sarcoidosis, Interferon, Interferon stimulated gene, Uveitis

## Abstract

**Objectives:**

In this study, we measured serum interferon (IFN) levels and activity in patients with sarcoidosis and tuberculosis (TB) with and without uveitis. We aimed to understand the role of IFN in the pathophysiology of both conditions and explore its potential as a discriminating marker for these clinically similar diseases.

**Methods:**

Sera from an Indonesian TB and a Dutch sarcoidosis cohort were used in the analysis. IFNα2 and IFNγ concentrations were measured using Simoa® and Luminex assays, respectively. Serum IFN activity was assessed by incubating THP-1 cells with patient serum and measuring IFN-stimulated gene transcription using qPCR. Anti-IFNα2 and IFNγ autoantibodies were detected via Luminex assay and tested for neutralizing capacity using a flow cytometry-based signal transducer and activator of transcription (STAT) 1 phosphorylation inhibition assay.

**Results:**

IFNα2 was detected in 74 % and 64 % of patients with sarcoidosis and pulmonary TB, respectively, while IFNγ was found in 78 % and 23 % of patients with sarcoidosis and TB, respectively. For uveitis cases specifically, IFNα2 was detected in 85 % of sarcoid uveitis (SU) and 33 % of tubercular uveitis (TBU) cases. Similarly, IFNγ was detected in 69 % of SU and 17 % of TBU cases. IFNγ serum concentrations were higher in sarcoidosis than that in TB patients (*p* < 0.0001). Focusing on patients with uveitis, SU showed increased IFNα2 (*p* = 0.004) and IFNγ (*p* < 0.002) serum concentrations compared to that in TBU. Notably, TBU displayed significantly reduced IFNα2 concentrations compared to that in healthy controls (*p* = 0.006). These results align with the increased interferon stimulated gene (ISG) transcriptional upregulation observed in THP-1 cells stimulated with serum from patients with sarcoidosis. Elevated levels of non-neutralizing anti-IFN autoantibodies were observed in patients with TB; however, these levels were similar to those observed in geographically matched healthy Indonesian controls.

**Conclusion:**

Our results suggest decreased serum levels and activity of type I and II IFN in TB compared to those in sarcoidosis. This is indicative of distinct pathophysiological processes in these highly clinically similar diseases. We propose that the assessment of serum IFN levels and IFN activity has the potential to distinguish between sarcoidosis/SU and TB/TBU.

## 1 Introduction

Sarcoidosis is a systemic granulomatous inflammatory disease with an unclear etiology, likely involving environmental antigens and genetic factors (e.g., HLA alleles and NOD2 variants) that trigger an abnormal immune response and granuloma formation [1]. Tuberculosis (TB), caused by *Mycobacterium tuberculosis* (*Mtb*), leads to granuloma formation due to ineffective pathogen clearance [2,3]. Sarcoidosis and TB share overlapping clinical features, commonly affecting the lungs and thoracic lymph nodes, but can involve any organ, including the eyes [1,2]. Ocular involvement occurs in 10–80 % of sarcoidosis cases, with 20–30 % attributed to uveitis (sarcoid uveitis: SU) [[4], [5], [6], [7], [8]]. Tubercular uveitis (TBU) occurs in 1.4–18 % of pulmonary TB cases [9,10].

The clinical overlap between sarcoidosis and TB suggests a potential shared pathogenesis, and substantial overlap between sarcoidosis and TB gene and microRNA signatures within blood samples, lymph node tissue, and lung tissue has been reported [[11], [12], [13], [14]]. Some researchers have hypothesized that sarcoidosis and TB represent opposite ends of the same disease spectrum [[15], [16], [17]]. The similarity between the two diseases also poses a diagnostic challenge in discriminating between sarcoidosis and TB, especially in cases of isolated SU and TBU. Therefore, better insight into disease pathogenesis is required, as this may result in improved diagnostic options [[18], [19], [20]].

Interferons (IFNs) play important roles in the immunopathogenesis of sarcoidosis and TB. In patients with sarcoidosis, elevated numbers of IFNγ-producing CD4^+^ T lymphocytes have been identified in bronchoalveolar lavage (BAL) fluid, peripheral blood, and mediastinal lymph nodes [[21], [22], [23], [24]]. Moreover, elevated IFNγ (type II IFN) inducible protein 10 (IP-10/CXCL10) and higher cellular expression of the transcription factor T-bet, which regulates IFNγ expression, have been noted in BAL samples [[25], [26], [27]]. Also, IFNγ-producing Th1 and Th17.1 lymphocytes accumulate in sarcoid granulomatous tissue [28,29]. Detectable levels of IFNγ in the serum of patients with sarcoidosis further underscores the involvement of IFNγ in this disease [30]. Besides IFNγ, a recent meta-analysis conducted on gene-expression studies from several different tissues revealed that type I IFN mediated signaling pathways are also prominently involved in sarcoidosis [31]. Consistent with this, IFN type I treatment for hepatitis C is associated with an increased risk of sarcoidosis, similar to certain *IFNA* polymorphisms [[32], [33], [34], [35], [36]].

The protective role of IFNγ in controlling an *Mtb* infection is well-documented in animal models and human studies. IFNγ enhances intracellular control of *Mtb* by induction of antimicrobial activity in macrophages, including the production of reactive oxygen species [[37], [38], [39]]. Consistently, molecular defects in IFNγ related pathways underlie Mendelian susceptibility to mycobacterial diseases [[40], [41], [42]]. In addition, autoantibodies against IFNγ increase the risk of infection with nontuberculous mycobacteria and *Mtb* [43,44]. Although not fully understood, type I IFNs likely play a protective role early during *Mtb* infection by initiating host-protective immune responses [45]. However, effective *Mtb* control requires a rapid shift towards a protective IFNγ-driven immune response. In contrast, chronic type I IFN production impairs a Th1 response and reduces survival rates in *Mtb*-infected mice. Furthermore, *in vitro* human cell experiments showed that type I IFN negatively affects the production of IL-1α, IL-1β, IFNγ, and IL-12, which are crucial for controlling an *Mtb* infection [[45], [46], [47], [48]]. These effects contribute to the ineffective control of *Mtb* and may thereby facilitate the dissemination of *Mtb*, potentially affecting other organs, including the eyes [45,49]. However, type I IFN may be beneficial in specific cases. In refractory TB and in patients with defective IFNγ receptor signaling, co-administration of IFNα with antitubercular treatment led to clinical improvement [45,49]. Similarly, murine models showed beneficial effects of type I IFN in the absence of IFNGR signaling, resulting in reduced lung pathology and improved control of bacterial replication [45]. Thus, early production of type I IFN, preceding the IFNγ response, might be crucial for the induction of an appropriate immune response (including: recruitment, differentiation, and survival of myeloid cells that control early infection, as well as the induction of dendritic cell maturation, and associated IL-12 production and T-lymphocyte priming), while a more chronic IFN type I response might increase disease susceptibility [50].

Although type I, II, and III IFNs use different receptors, their downstream JAK-STAT signaling pathways overlap, resulting in the induction of IFN-stimulated genes (ISGs) [51]. Enrichment of ISG expression was observed in the peripheral blood cells of individuals with active TB and sarcoidosis [14,[52], [53], [54], [55]]. Maertzdorf et al. found overlapping peripheral blood transcriptional profiles between patients with active pulmonary sarcoidosis and TB [14], while Zhao et al. identified IFNγ signaling in lung tissue and peripheral blood from both pulmonary sarcoidosis and TB [56], suggesting that systemic IFN circulation is involved in ISG induction. Notably, Llibre et al. reported a discordance between increased peripheral blood ISG expression and serum IFNα levels, with undetectable serum IFN activity in patients with TB [57]. Furthermore, Mulenga et al. reported a weak inverse correlation between an 11-gene IFN transcriptional signature (RISK11) and the capacity of peripheral blood cells from patients with TB to produce IFNγ upon *in vitro* exposure to TB antigens [58]. Alternatively, studies report increased serum IFNγ in active TB disease compared to that in healthy controls (HCs) [14,[59], [60], [61]], although other studies did not observe this relation between active TB disease and increased serum IFNγ concentrations [[62], [63], [64], [65]].

Despite the clinical and pathological similarities between sarcoidosis and TB, there is no consensus on the role of circulating IFNs in these conditions. Moreover, direct comparisons of the circulating IFN levels in these diseases are rare. Two studies described identical IFNγ serum concentrations in TB and sarcoidosis, but this was not explored in relation to uveitis [64,65]. Therefore, further research on circulating IFNs in sarcoidosis and TB is warranted to reveal the differences and similarities in their immune pathogenesis. Moreover, whether circulating IFN can serve as a diagnostic biomarker to differentiate sarcoidosis from TB, especially in cases of isolated uveitis, remains of interest because of current diagnostic challenges [17,66,67].

In the current study, we aimed to gain a deeper insight into the involvement of IFN in TB and sarcoidosis. Therefore, we used highly-sensitive immunoassays and bioassays to measure IFNα2, IFNγ, IFN activity, and IFN-autoantibodies in serum from patients with active TB and sarcoidosis, with and without uveitis, and additionally explored potential diagnostic utility.

## 2 Methods

### 2.1 Patients and controls

Sera from a previously described Indonesian TB cohort (cohort 1) and our local Dutch Sarcoidosis Biobank cohort (Departments of Internal Medicine and Immunology, Erasmus MC; Cohort 2) was used in this study [[68], [69], [70]]. Cohort 1 consisted of 22 treatment-naïve patients with active pulmonary TB, of which 12 had TBU and 23 Indonesian HCs [70]. Cohort 2 consisted of 27 patients with sarcoidosis, 13 of whom had SU. None of the patients with sarcoidosis had received any immunomodulatory medication in the three months prior to blood sampling. Detailed patient information is provided in Supplementary Table 1. In addition, available serum from 19 healthy Caucasian controls was included, which could only be used in a number of the assays conducted owing to the limitations of the available material.

The study was approved by the local medical ethics committee of the Faculty of Medicine, University of Indonesia (cohort 1, FMUI: 268/H2.F1/ETIK/2014), and Erasmus MC, University Center Rotterdam (MEC-2014-476, MEC-2020-0193, and MEC-2016-202) and conducted in accordance with the tenets of the Declaration of Helsinki.

### 2.2 IFNα2 measurement using ultrasensitive single molecule detection array

IFNα2 was measured in duplicates from two-fold diluted serum samples by the ultra-sensitive magnetic bead single molecule detection array Simoa® platform using the IFNα Advantage Kit (no. 100860, Quanterix, Billerica, MA, USA), according to manufacturer's instructions. Sample processing and analysis were performed using an HD-X analyzer (software version June 1, 1905.300; Quanterix). Samples in which IFNα2 concentration could not be calculated by extrapolation were put at half of the lowest extrapolated value (0.05 fg/mL). Previously, we demonstrated the high specificity of this test for detecting IFNα2, but not the other 12 IFNα subtypes, IFNβ or IFNγ [71].

### 2.3 IFNγ measurement using a bead-based immunoassay

IFNγ levels were determined in two-fold diluted serum samples with a Luminex bead-based high performance assay (LUXLM285; R&D Systems Europe, Abingdon, UK). The assay was performed according to the manufacturer's instructions. Samples for which the IFNγ concentration could not be calculated by extrapolation were placed at half the lowest extrapolated value (0.075 pg/mL).

### 2.4 Functional testing for serum IFN activity

The human monocytic leukemia cell line, THP-1, was cultured in Roswell Park Memorial Institute Medium (RPMI; Gibco, Grand Island, NY, USA) supplemented with 10 % fetal calf serum (FCS) and antibiotics (penicillin and streptomycin; BioWhittaker, Verviers, Belgium) in a humidified incubator at 37 °C and 5 % CO_2._

THP-1 cells were seeded in duplicates for each stimulation condition at a density of 1.5 × 10^5^ cells/well into 96-well plates (Thermo Fisher Scientific, Waltham, MA, USA) in an initial volume of 50 μL RPMI supplemented with 10 % FCS and antibiotics (culture medium). Hereafter, 50 μL of plane culture medium (negative control), 50 μL of culture medium supplemented with either recombinant human (rh)IFNγ, rhIFNβ (both from Peprotech, London, UK), or rhIFNα (Sigma, Saint Louis, MO, USA) at a final concentration of 5 ng/mL (positive controls) or 50 μL of patient serum was added to the cells. After an incubation period of 3 h (optimal time point for ISG induction as established by initial time-course experiments where THP-1 cells were stimulated with rhIFNγ, rhIFNβ, or rhIFNα over a time course of 30 min to 24 h; data not shown) the THP-1 cell suspensions were collected and culture duplicates were pooled. Thereafter, cell suspensions were pelleted (500 Relative Centrifugal Force (RCF) for 5 min) and washed with 500 μL PBS (pH 7.4). Then, RNA was isolated with the GenElute™ Mammalian Total RNA Miniprep Kit (Sigma) and quantified with the use of the NanoDrop™ One Microvolume UV–Vis Spectrophotometer (Thermo Fisher Scientific). Complementary DNA was synthesized from 1 μg of RNA and the expression level of ten ISGs (*TLR8*, *FCGR1B*, *GBP1*, *IFIT2*, *IRF7*, *MYD88*, *SERPING1*, *STAT1*, *UBE2L6,* and *MX1*) was determined through normalization against the reference gene *ABL1*. These ten ISGs were selected based on our previous studies where we found that their expression levels in peripheral blood cells were associated with TB [52]. Primer-probe combinations were obtained from Thermo Fisher Scientific (Supplementary Table 2).

A composite IFN signature score (∑ (ΔC_t(stimulated)_ – Mean ΔC_t(unstimulated)_) *s ΔC_t(unstimulated)_^−1^) was calculated from the individual gene expression levels as described previously [52]. The ten ISGs were each assigned a ΔΔCt value that was calculated by subtracting the ΔCt value of each gene obtained in THP-1 cells upon stimulation with serum from an individual (patient with TB or sarcoidosis, designated as stimulated in the formula) from the average ΔCt value of that gene obtained in THP-1 cells upon stimulation with serum from HCs (designated as unstimulated in the formula). This was then divided by the standard deviation of the ΔCt values of that gene in the HC group. The IFN signature score for the entire gene set was determined by summing these individual gene scores (ΔΔCt values).

### 2.5 Anti-IFNα2 and anti-IFNγ autoantibody measurement using a bead-based immunoassay

Anti-IFNα2 and anti-IFNγ autoantibodies were measured in 1:100 diluted serum samples using recombinant human cytokine-coupled beads. Sera from patients with autoimmune polyendocrinopathy candidiasis ectodermal dystrophy (APECED) and adult-onset immunodeficiency (AOID) with high levels of pathogenic anti-IFNα autoantibodies or anti-IFNγ autoantibodies were used as positive control samples, as described previously [71,72].

Recombinant human IFNα2 (PBL Assay Science; 11102-2) and rhIFNγ (R&D; 285-IF-100/CF) were conjugated to MC10029–01 and MC10027-01 Magplex beads (Luminex), respectively and according to a standard protocol for protein coupling as supplied by Luminex (https://info.diasorin.com/en-us/research/download-the-xmap-cookbook). To measure anti-IFN autoantibodies, IFN-coupled beads were brought to room temperature (RT), vortexed for 30 s, sonicated for 30 s, and vortexed for another 30 s. Then the beads were diluted in PBS/BSA 0.5 % to a final concentration of 5 × 10^4^ beads per mL, of which 50 μL was added to individual reaction wells in a Bioplex Pro Flat Bottom 96-well Plate (Bio-Rad, Hercules, CA, USA). Serum samples from patients, HCs, and positive control sera (for anti-IFNα2 from patient with APECED and anti-IFNγ from a patient with AOID) were diluted 1:100 in PBS/BSA 0.5 % and 50 μL was added to the designated wells after which the plate was incubated at RT for 3 h on a titer plate shaker (Thermo Fisher Scientific). Thereafter, the plate was washed using a magnetic microtiter plate washer (Bio-Rad) and 100 μL of goat anti-human PE (Bioconnect, Huissen, the Netherlands 109-116-098, dilution 1:100 in PBS/BSA 0.5 %) was added to the wells. The plate was then incubated at RT for 30 min on a titer plate shaker and washed with a magnetic microtiter plate washer. Hereafter, the beads were resuspended in 100 μL PBS, incubated at RT for 5 min on the titer plate shaker and measured on the Bio-Plex Magpix (Luminex) yielding mean fluorescence intensity (MFI) values.

### 2.6 Functional testing of anti-IFN autoantibody activity

To investigate whether anti-IFNα and anti-IFNγ autoantibodies were neutralizing, inhibition of IFNα or IFNγ induced signal transducer and activator of transcription (STAT) 1 phosphorylation in THP-1 cells was assessed. Sera from a subset of 12 patients with the highest serum levels of anti-IFN autoantibodies (TB, n = 3; TBU, n = 3; sarcoidosis, n = 3; and SU, n = 3) were selected.

THP-1 cells suspended at a density of 6 × 10^6^/mL in 80 μL PBS were incubated with 10 ng/mL IFNγ (Peprotech) or 100 ng/mL IFNα (Merck, Rahway, NJ, USA) that was either pre-incubated for 10 min at 37 °C with 5 μL serum or not. Cells were incubated for 30 min (optimal time point to measure IFNγ and IFNα induced STAT-1 phosphorylation in THP-1 cells as deduced from initial assays over a time course of 5 min to 1 h; data not shown) at 37 °C after which 2 mL of diluted Lyse/Fix (Becton Dickinson [BD], Franklin Lakes, NJ, USA) was added for an additional 10 min at 37 °C. Cells were pelleted washed with ice-cold wash buffer (PBS, pH 7.4, 0.5 % FCS), resuspended in 500 μL Perm Buffer II (BD) and incubated for 12 min on ice. Subsequently, the cells were washed with ice-cold wash buffer, stained with anti-pSTAT1-AF488 (Cell Signaling Technology, Danvers, MA, USA) and anti-STAT1-PE (Cell Signaling Technology) for 30 min at RT, and shielded from light. After washing, cells were analyzed using a BD FACSLyric Cell Analyzer. Flow cytometric data were processed using the Infinicyt 2.0.6.b.001 software (Cytognos, Salamanca, Spain). All washing steps were performed at 500 RCF for 5 min.

### 2.7 Statistical analysis

Statistical analyses were performed using GraphPad Prism 9.2.0. Multiple group comparisons were made using the Kruskal–Wallis test, followed by post hoc Dunn's multiple comparisons. Comparisons between two groups were made using the Mann–Whitney *U* test, and correlations were determined using Spearman's rank correlation coefficient. A *p* value of ≤0.05 was considered statistically significant. Youden's indices were only reported above 0.50, as a value below 0.50 does not meet the empirical standards to contribute as a potential diagnostic test.

## 3 Results

### 3.1 Serum IFNα2 concentrations determined by ultrasensitive single molecule detection array

Detectable levels of IFNα2 were present in 14 out of 22 (64 %) serum samples from patients with TB, 20 out of 27 (74 %) patients with sarcoidosis and 28 out of 32 (88 %) HCs (Fig. 1A). No significant difference in serum IFNα2 concentration was observed between pulmonary TB (median = 4.4 fg/mL), sarcoidosis (median = 20.6 fg/mL), and HCs (median = 17.4 fg/mL; Fig. 1A). HCs from Indonesia displayed higher IFNα2 serum concentrations (median = 22.3 fg/mL; *p* < 0.001) than Caucasian HCs (median 9.9 fg/mL; Fig. 1A).

**Fig. 1:**
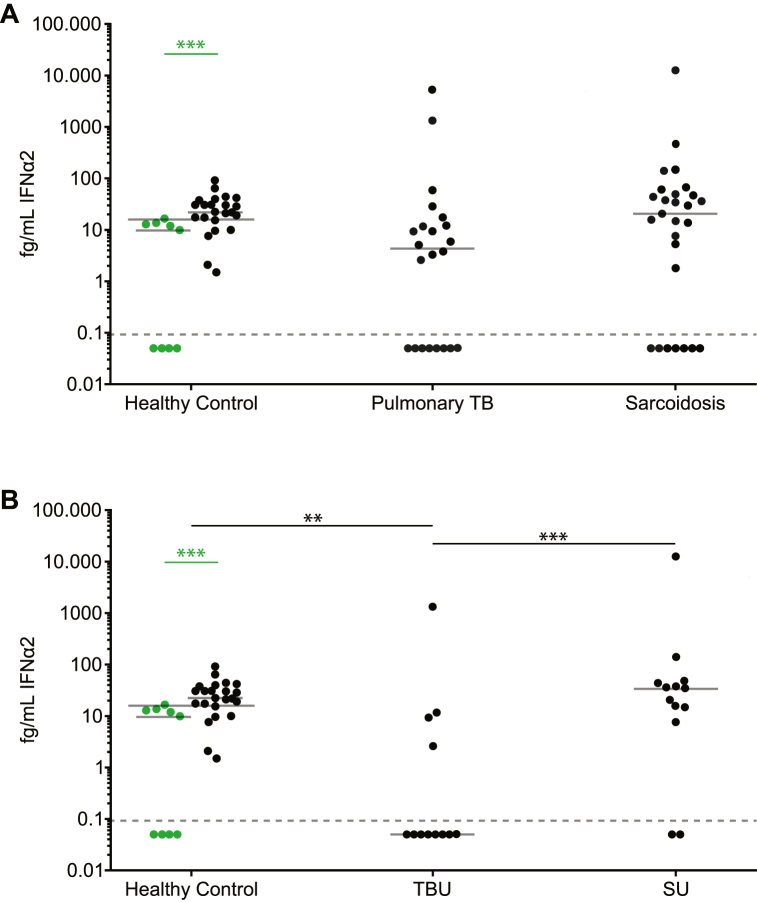
Serum interferon (IFN)α2 levels, as measured by ultra-sensitive single-molecule digital enzyme-linked immunosorbent assay (ELISA) IFNα2 levels were measured in 10 patients with pulmonary tuberculosis (TB) without uveitis, 12 patients with tubercular uveitis (TBU), 14 patients with sarcoidosis without uveitis, 13 patients with sarcoid uveitis (SU), 23 Indonesian healthy controls and 9 Caucasian healthy controls. **(A,B)** Increased serum IFNα2 levels were detected in Indonesian healthy controls as compared to that in Caucasian controls. **(B)** Decreased levels of IFNα2 were detected in patients with TBU as compared to that in patients with SU and healthy controls. Green dots represent healthy Caucasian controls. ** = *p* < 0.01, *** = *p* < 0.001. Solid gray lines indicate the median, and the gray dotted line indicates the lowest extrapolatable value. Statistical analysis was performed using GraphPad Prism 9.2.0. A Kruskal–Wallis test followed by Dunn's multiple comparisons test was used to compare groups, and a Mann–Whitney *U* test was used to compare two groups of healthy controls. (For interpretation of the references to colour in this figure legend, the reader is referred to the Web version of this article.)

Regarding uveitis, positivity for serum IFNα2 was more frequently observed in SU (11/13 patients: 85 %; *p* = 0.015; Fisher's exact test) than TBU (4/12 patients: 33 %; Fig. 1B). Although no statistical significant difference in serum IFNα2 concentration was observed between total TB and sarcoidosis groups (Fig. 1A), patients with TBU had significantly lower serum IFNα2 concentrations (median = 0.05 pg/mL) than that in patients with SU (median = 34.70 pg/mL; *p* = 0.004; Fig. 1B). Serum IFNα2 concentration in the TBU group was also significantly lower than in the HC group (median = 0.05 pg/mL vs median = 17.4 fg/mL, respectively; *p* = 0.006; Fig. 1B).

Next we calculated the diagnostic potential of serum IFN-α2 to discriminate between patients with SU and TBU. This comparison resulted in a maximum Youden's index of 0.69 with a corresponding sensitivity of 77 % and specificity of 92 % at a cut-off concentration of 13.25 pg/mL.

### 3.2 Serum IFNγ concentrations determined by bead-based immunoassay

Detectable levels of IFNγ were more frequently observed in patients with sarcoidosis (21/27:78 %) than patients with TB (5/22:23 %; *p* < 0.0001; Fisher's exact test) and HCs (2/32:6 %; *p* < 0.0001; Fisher's exact test) (Fig. 2A). Serum IFNγ concentrations did not differ between Indonesian and Caucasian HCs (Fig. 2A). Serum IFNγ concentrations were significantly (*p* < 0.001) higher in patients with sarcoidosis (median = 2.760 pg/mL) than in those with TB (median = 0.075 pg/mL) and HCs (median = 0.075 pg/mL; Fig. 2A).

**Fig. 2:**
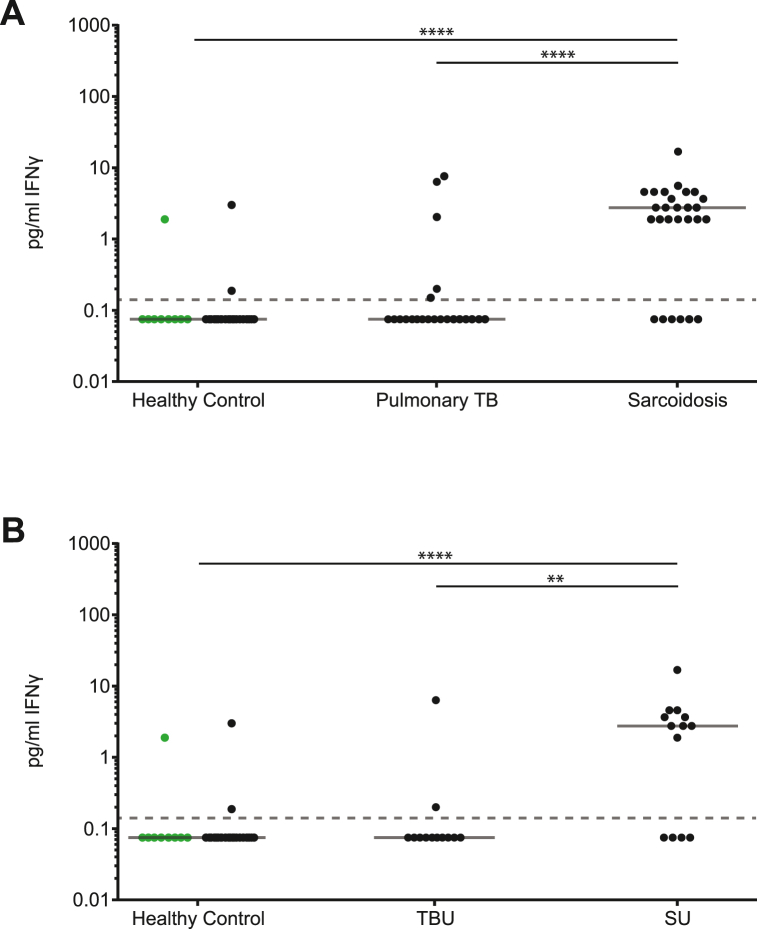
Serum IFNγ levels as measured by Luminex bead based assay IFNγ levels were measured in 10 patients with pulmonary TB without uveitis, 12 patients with pulmonary TBU, 14 patients with sarcoidosis without uveitis, 13 patients with SU, 23 Indonesian healthy controls, and 9 Caucasian healthy controls. **(A)** Increased serum IFNγ levels were detected in patients with sarcoidosis as compared to that in healthy controls and patients with pulmonary TB. **(B)** Furthermore, increased serum IFNγ levels were detected in SU as compared to that in healthy controls and patients with TBU. Green dots represent Caucasian healthy controls. ** = *p* < 0.01, **** = *p* < 0.0001. Solid gray lines indicate the median, and the gray dotted line indicates the lowest extrapolatable value. Statistical analysis was performed using GraphPad Prism 9.2.0. A Kruskal–Wallis test followed by Dunn's multiple comparisons test was used to compare groups, and a Mann–Whitney *U* test was used to compare two groups of healthy controls. (For interpretation of the references to colour in this figure legend, the reader is referred to the Web version of this article.)

In uveitis cases only, IFNγ positivity was observed more frequently in SU (9/13:69 %) compared to TBU (2/12:17 %; *p* = 0.015; Fisher's exact test; Fig. 2B). SU had a significantly higher serum IFNγ concentration (median, 2.760 pg/mL) than that of TBU (median, 0.075 pg/mL; *p* = 0.0031; Fig. 2B).

Serum IFN-γ discriminated between pulmonary TB and sarcoidosis with a maximum Youden's index of 0.64 and corresponding sensitivity of 78 % and specificity of 86 % specificity at a cut-off concentration of 1.045 pg/mL. Serum IFNγ discriminated between SU and TBU with a maximum Youden's index of 0.61 and corresponding sensitivity of 69 % and specificity of 92 % at a cut-off concentration of 1.045 pg/mL.

### 3.3 ISG induction in THP-1 cells

Next, we conducted a functional assay to explore the capacity of sera from TB, sarcoidosis, and HCs to induce the expression of 10 previously identified ISGs that were elevated in the blood cells of patients with active TB in THP-1 cells [52]. Serum from patients with sarcoidosis significantly (*p* < 0.01) induced the expression of *FCGR1B*, *GBP1*, *IFIT2*, *IRF7*, *MYD88*, *SERPING1*, *STAT1*, *UBE2L6,* and *MX1* compared to serum from patients with TB (Supplementary Figs. 1B–J). Compared to HCs, serum from patients with sarcoidosis significantly (*p* < 0.05) induced the expression of *FCGR1B* and *IFIT2*, whereas serum from patients with TB significantly (*p* < 0.05) reduced the expression of *GBP1*, *SERPING1,* and *UBE2L6* compared to HCs (Supplementary Fig. 1B–D,G,I). The induced expression levels of the individual genes *FCGR1B*, *GBP1*, *IFIT2*, *SERPING1,* and *UBE2L6* could discriminate between patients with sarcoidosis and TB with a maximum Youden's index >0.80 and corresponding sensitivity >88 % and specificity >89 % (Table 1).

**Table 1:** Diagnostic potential of individual interferon stimulated genes and interferon signature score.

Gene	Youden's index (All patients with SAR and TB/Only patients with uveitis)		Sensitivity (All patients with SAR and TB/Only patients with uveitis)	Specificity(All patients with SAR and TB/Only patients with uveitis)
*TLR8*	0.39/0.54		44/54	95/100
*FCGR1B*	0.88/0.92		93/92	95/100
*GBP1*	0.91/0.92		96/100	95/92
*IFIT2*	0.86/0.84		96/92	90/92
*IRF7*	0.58/0.69		63/77	95/92
*My88*	0.69/0.76		74/85	95/92
*SERPING1*	0.84/0.85		89/85	95/100
*STAT1*	0.66/0.76		81/92	85/83
*UBE2L6*	0.88/0.85		93/85	95/100
*MX1*	0.75/0.76		85/85	90/92
IFN sig. score	0.74/0.85		74/85	100/100

SAR = sarcoidosis, TB = tuberculosis, IFN = interferon, sig. = signature**,***TLR8* = toll like receptor 8, *FCGR1B* = Fc fragment of IgG receptor Ib, *GBP1* = guanylate binding protein 1, *IFIT2* = interferon induced protein with tetratricopeptide repeats 2, *IRF7* = interferon regulatory factor 7, *My88* = myeloid differentiation primary response 88, *SERPING1* = serpin family G member 1, *STAT1* = signal transducer and activator of transcription 1, *UBE2L6* = ubiquitin/ISG15-conjugating enzyme E2 L6, *MX1* = MX dynamin like GTPase 1.

A comparison of uveitis cases displayed the same findings, with significantly (*p* < 0.05) higher transcriptional induction of *FCGR1B*, *IFIT2*, *IRF7*, *MYD88*, *SERPING1*, *STAT1*, *UBE2L6,* and *MX1* in SU than in TBU (Supplementary Figs. 1A–J). Compared to HCs, sera from patients with SU significantly (*p* < 0.05) induced the expression of *FCGR1B* and *UBE2L6*, whereas sera from those with TBU significantly (*p* < 0.05) reduced the expression of *GBP1* and *SERPING1* (Supplementary Figs. 1B,C,G, and I). The expression levels of the individual genes *FCGR1B*, *GBP1*, *IFIT2*, *SERPING1,* and *UBE2L6* discriminated between SU and TBU with a maximum Youden's index >0.80, and corresponding sensitivity >84 % and specificity >90 % (Table 1).

**Fig. 3:**
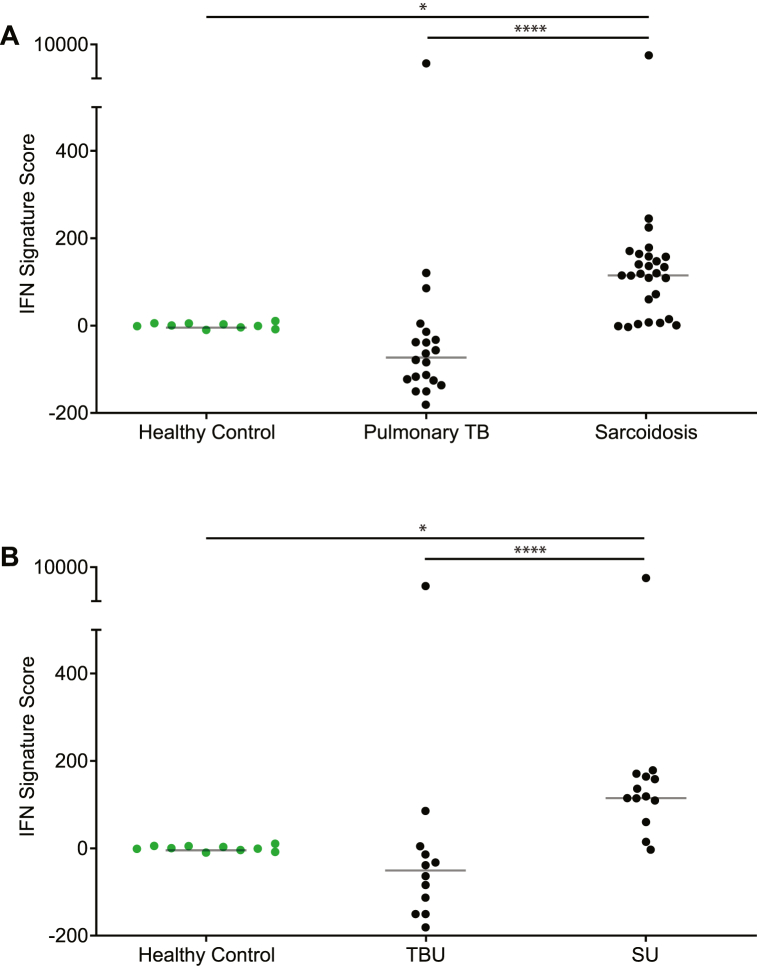
Ten gene based calculated IFN-signature score Calculated (∑ (ΔCt_(stimulated)_ – Mean ΔCt_(unstimulated)_) *s ΔCt_(unstimulated)_^−1^) IFN signature score according to 10 determined IFN stimulated gene (*TLR8, FCGR1B, GBP1, IFIT2, IRF7*, *My88, SERPING1, STAT1, UBE2L6,* and *MX1*) transcriptional levels after stimulation of the human monocytic leukemia cell line cell line THP-1 with serum derived from 8 patients with pulmonary TB without uveitis, 12 patients with pulmonary TBU, 14 patients with sarcoidosis without uveitis, 13 patients with SU and 10 Caucasian healthy controls. **(A)** An increased IFN signature score was detected in patients with sarcoidosis compared to that in healthy controls and patients with pulmonary TB. **(B)** An increased IFN signature score was detected in patients with SU compared to that in healthy controls and patients with TBU. Green dots represent Caucasian healthy controls. * = *p* < 0.05, ** = *p* < 0.01, **** = *p* < 0.0001. The solid gray lines indicate the median values. Statistical analysis was performed using GraphPad Prism 9.2.0, and the Kruskal–Wallis test followed by Dunn's multiple comparisons test was used to compare the groups. (For interpretation of the references to colour in this figure legend, the reader is referred to the Web version of this article.)

Next, an integrated IFN gene signature score was calculated from the serum-induced expression levels of the 10 individual ISGs. Although not statistically significant, a trend towards a reduced IFN-gene signature was observed in patients with TB compared to that in HCs (Fig. 3A). In contrast, serum from sarcoidosis resulted in a significantly (*p* < 0.01) higher IFN-gene signature score than that from patients with pulmonary TB and HCs (Fig. 3A). Also, for uveitis cases only, serum from SU induced a significantly (*p* < 0.01) higher IFN-gene signature score in THP-1 cells than serum from patients with TBU (Fig. 3B). Again, a trend towards a reduced IFN-gene signature in TBU compared to that in HCs was observed (Fig. 3B).

Finally, we determined the diagnostic potential of the serum-induced IFN gene signature score for discriminating between pulmonary TB and sarcoidosis. At a maximum Youden's index of 0.74, a corresponding sensitivity of 74 % and specificity of 100 % were achieved at an IFN-gene signature cutoff score of 55.44. When only patients with sarcoidosis and TB with uveitis were examined, a maximum Youden's index of 0.85 resulted in 85 % sensitivity and 100 % specificity to discriminate TBU from SU at a serum-induced IFN-gene signature cut-off score of 52.05.

### 3.4 Detection of anti-IFN autoantibodies

**Fig. 4:**
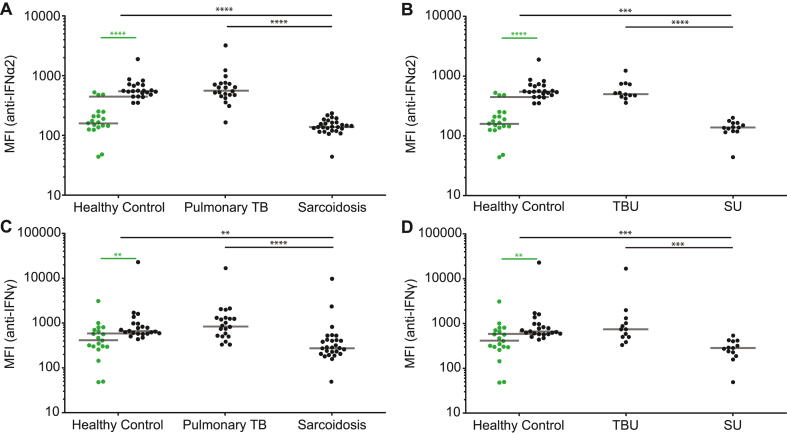
Anti-IFNα2 and anti-IFNγ autoantibodies Serum **(A,B)** anti-IFNα2 and **(C,D)** anti-IFNγ autoantibodies as measured using a Luminex bead based assay in 9 patients with pulmonary TB without uveitis, 12 patients with pulmonary TBU, 14 patients with sarcoidosis without uveitis, 13 patients with SU, 22 Indonesian healthy controls, and 19 Caucasian healthy controls. **(A**–**D)** Increased serum anti-IFNα2 and anti-IFNγ autoantibody levels were detected in Indonesian healthy controls as compared to those in Caucasian healthy controls. **(A, C)** Patients with pulmonary TB displayed increased levels of anti-IFNα2 and anti-IFNγ autoantibodies as compared to those in patients with sarcoidosis; however, these were at a similar levels as geographically matched healthy controls. **(B,D)** Furthermore increased serum anti-IFNα2 and anti-IFNγ autoantibody levels were detected in patients with TBU as compared to those in patients with SU; however, these were at a similar levels as geographically matched healthy controls. Green dots represent healthy Caucasian controls. ** = *p* < 0.01, *** = *p* < 0.001, **** = *p* < 0.0001. The solid gray lines indicate the median values. Statistical analysis was performed using GraphPad Prism 9.2.0. A Kruskal–Wallis test followed by Dunn's multiple comparisons test was used to compare groups, and a Mann–Whitney *U* test was used to compare two groups of healthy controls. MFI = mean fluorescence intensity. (For interpretation of the references to colour in this figure legend, the reader is referred to the Web version of this article.)

Hardly any IFN immunoreactivity and even a trend towards decreased bioactivity was observed in the circulation of patients with TB, therefore, we examined whether autoantibodies against IFNα2 and IFNγ could be detected in the serum samples. Anti-IFNα2 and anti-IFNγ autoantibody levels were significantly (*p* < 0.0001) higher in Indonesian HCs than in Caucasian HCs (Fig. 4A–C). Furthermore, patients with TB had significantly (*p* < 0.0001) higher anti-IFNα2 and anti-IFNγ autoantibody levels than in patients with sarcoidosis; however, these concentrations were comparable to Indonesian HCs (Fig. 4A–C). Anti-IFNα2 and anti-IFNγ autoantibody levels also did not differ between patients with sarcoidosis and Caucasian HCs (Fig. 4A–C). Comparison of patients with uveitis only revealed comparable results with significantly (*p* < 0.001) higher serum anti-IFNα2 and anti-IFNγ antibody levels in TBU compared to those in SU; these concentrations were again comparable to Indonesian HCs (Fig. 4B–D). Observed levels of anti-IFNα2 autoantibodies in HCs, sarcoidosis, and TB were substantially lower compared to the level of anti-IFNα2 observed in a patient with APECED (MFI = 29380) and anti-IFNγ in a patient with AOID (MFI = 33381) as detected at a 1000-fold serum dilution. The levels of anti-IFNα2 and anti-IFNγ autoantibodies showed no correlation with the serum concentrations of IFNα2 or IFNγ nor with the interferon activity detected in the serum samples of either the TB or sarcoidosis group (Supplementary Figs. 2A and B).

### 3.5 Neutralizing capacity of serum derived from patients with sarcoidosis or TB

In contrast to the APECED serum (containing high concentrations of IFNα neutralizing autoantibodies), we could not detect an inhibitory effect of serum from TB (n = 6) or sarcoidosis (n = 6) with high anti-IFNα2 reactivity on IFNα induced STAT1 phosphorylation in THP-1 cells (Supplementary Fig. 3A). In contrast to the adult-onset immunodeficiency serum (containing high concentrations of IFNγ neutralizing autoantibodies), serum from patients with TB (n = 6) and three (n = 1 SU and n = 2 sarcoidosis without uveitis) out of six sera from patients with sarcoidosis did not inhibit IFNγ-induced STAT1 phosphorylation in THP-1 cells (Supplementary Fig. 3B).

## 4 Discussion

Here, we provide evidence that sarcoidosis is associated with elevated systemic (serum) IFN levels and activity (including type I and II IFNs); however, this is not the case in TB. This highlights differences in the immune pathogenesis of both diseases and suggests that measurement of serum IFNα2, IFNγ and/or IFN-activity may have diagnostic potential in discriminating both diseases.

Gene transcriptional signatures highly enriched for ISGs in peripheral blood and other tissues have been described in active TB and sarcoidosis, and proposed as biomarkers to discriminate between active and latent *Mtb* infections and monitor treatment response [[52], [53], [54], [55],^73^]. However, owing to the significant overlap in ISG signatures between active TB and sarcoidosis, ISG measurement in the peripheral blood seems insufficient to discriminate between both diseases [14,53]. Notably, Llibre et al. noted that, in TB, an increase in blood cell ISG expression occurred in the absence of detectable serum IFN activity, with no or low detectable serum IFNα levels [57]. However, the study by Llibre et al. made use of a laboratory developed Simoa assay with low sensitivity for IFNα2 [57]. In our study, we used a commercial Simoa assay that is highly specific for IFNα2 [71] but found no evidence of elevated circulating IFNα2 in TB. Data on circulating IFNγ levels in patients with TB are inconsistent [14,[58], [59], [60], [61], [62], [63], [64], [65]]. In our current study we used several approaches to explore circulating IFNγ, yet found no evidence for elevated IFNγ levels nor activity in TB. Our data indicate that TB, in contrast to sarcoidosis, is not associated with systemic increased concentrations of IFNα, IFNγ, and IFN-activity. Our data are consistent with those of Libre et al. and support their hypothesis that the ISG profiles identified in the circulating blood cells of patients with TB are likely induced in anatomical compartments distinct from the peripheral blood, presumably at sites of infection or within draining lymphoid tissues [57] and further indicate pathophysiological differences between TB and sarcoidosis.

Uveitis can develop in the context of sarcoidosis or TB, and can even be the main site of disease without significant clinical disease in other organ systems [74,75]. In this study, we analyzed the relationship between IFNs and uveitis in both sarcoidosis and TB. We observed that patients with TBU had reduced serum IFNα2 concentrations compared to that in TB cases without uveitis. We propose that a diminished capacity to mount a type I IFN response, which contributes to early anti-*Mtb* immunity [45,49], makes individuals more prone to *Mtb* dissemination from the lungs to other tissues, including the eyes. This aligns with our previous observation that peripheral blood cells from patients with active pulmonary TB and uveitis display lower ISG signatures than those from patients with active pulmonary TB without uveitis [52]. Nevertheless, ocular fluids from patients with TBU have been found to contain higher levels of the IFN-inducible chemokines CXCL9 and CXCL10 than ocular fluids from patients with idiopathic uveitis and HCs [[76], [77], [78], [79]]. These data highlight the role of local ocular IFN activity in TBU, which is further supported by the strong IFN-driven response observed in RPE cells infected with *Mtb*, along with the presence of *Mtb* reactive IFNγ-producing CD4^+^ T-lymphocytes in the vitreous humor of patients with TBU [80,81].

In contrast to TB, sarcoidosis was associated with increased serum IFNγ levels and both IFN-I and II activity, as well as elevated serum IFNα2 in SU compared to those in TBU. This data confirms earlier findings of elevated circulating IFNγ levels in sarcoidosis and is consistent with the increased serum levels of the IFN inducible chemokines, CXCL9 and CXCL10, reported in sarcoidosis [30,82,83]. Systemic IFN-I/II activity has been closely associated with the onset of autoimmune diseases [84] and a clear correlation has been established between elevated expression of ISGs in the peripheral blood and increased levels of circulating IFNs in systemic autoimmune disorders (e.g., systemic lupus erythematosus and Sjögren's syndrome) [71,[84], [85], [86], [87]]. Similarly, autoimmune features have been reported in sarcoidosis, including the presence of autoantibodies directed against Ro52, Ro60, SSB, Rib-P, CCP, and β2-glycoprotein [88]. Therefore, our data, along with findings from other studies, support the idea that elevated systemic IFN-I/II activity is involved in sarcoidosis and likely contributes to the increased expression of ISGs, as observed in peripheral blood mononuclear cells, BAL cells, and other affected tissues [73,83].

Clinical differentiation between sarcoidosis/SU and TB/TBU can be challenging, particularly in the context of isolated granulomatous uveitis [17]. Therefore, we explored whether circulating IFN concentrations and/or activity could serve as potential biomarkers to discriminate between sarcoidosis/SU and TB/TBU. In our study, serum IFNγ levels could discriminate between sarcoidosis and TB (Youden's index = 0.64, sensitivity = 78 %, specificity = 86 %, and AUC = 0.785) as well as between SU and TBU (Youden's index = 0.61, sensitivity = 69 %, specificity = 92 %, and AUC = 0.769). Additionally, serum IFNα2 showed discriminatory ability between SU and TBU (Youden's index = 0.69, sensitivity = 77 %, specificity = 92 %, and AUC = 0.821). The highest diagnostic accuracy for discriminating between sarcoidosis and TB was achieved by assessing serum IFN activity by measuring the induction of ten distinct ISG in THP-1 cells, and subsequently calculating an integrated IFN gene signature score (Youden's index = 0.74, sensitivity = 74 %, specificity = 100 %, and AUC = 0.900). This approach was optimal for discriminating between SU and TBU (Youden index = 0.85, sensitivity = 85 %, specificity = 100 %, and AUC = 0.897). Therefore, we propose that the serum measurement of IFN concentration and/or activity is of potential interest in discriminating between sarcoidosis/SU and TB/TBU. However, this should be validated in other studies with larger cohorts, especially due to existing conflicting data on the capacity of IFNγ to discriminate between sarcoidosis and TB [65]. Moreover, it is recommended to directly compare these measurements with other biomarkers that have been proposed to differentiate between sarcoidosis and TB, such as serum levels of leptin (AUC = 0.76), ICAM-1 (AUC = 0.72) C1q (AUC = 0.69), TNF-α (AUC = 0.95), IL-9 (AUC = 0.92), IL-10 (AUC = 0.80) and IL-17 (AUC = 0.94), especially in relation to uveitis [65,89,90].

Our study has several limitations. We conducted a single measurement, therefore, we were unable to evaluate the influence of chronicity or duration of the disease on our IFN measurements. We consider it unlikely that anti-IFNα2 or anti-IFNγ autoantibodies interfered with the detection of IFNα2 or IFNγ, due to the fact that we did not observe a correlation between anti-IFNα2 and anti-IFNγ autoantibody levels, and serum IFN levels or activity. Additionally, preincubation of IFNα or IFNγ with sera of patients with TB did not prevent induction of STAT1 phosphorylation in THP-1 cells. These observations suggested a lack of neutralizing activity of the detected autoantibodies [72]. However, we cannot exclude the possibility that anti-IFN autoantibody immune complexes were detected with the IFNα2 and IFNγ assays that we used, whereas our anti-IFN autoantibody assay failed to identify such complexes, which would lead to an underestimation of the level of anti-IFN autoantibodies present. In contrast, anti-IFN autoantibodies may have prevented IFN detection by the immunoassays we used because of epitope occupation or steric hindrance. Furthermore, we cannot exclude that other factors present in patient serum induced STAT1 phosphorylation and thereby masked a potential neutralizing effect of the anti-IFN autoantibodies in the experimental approach we used.

**Fig. 5:**
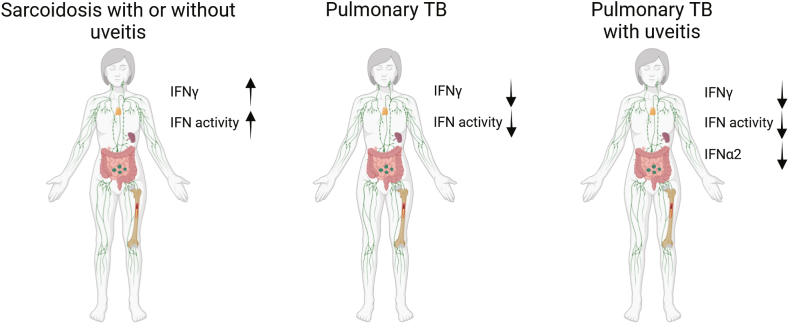
Increased serum IFN levels and activity in sarcoidosis compared to TB Schematic overview of our study results revealed diminished serum IFNγ levels and IFN activity in patients with TB and TBU in comparison to that in patients with sarcoidosis, both with and without uveitis. Furthermore, patients with TBU exhibited reduced serum IFNα2 levels compared to that in patients with sarcoidosis with and without uveitis, as well as patients with TB without uveitis (Created with BioRender.com).

In conclusion, our findings show clear differences between sarcoidosis and TB with respect to systemic IFN levels and activity. These findings underscore the differences in the pathophysiological processes between sarcoidosis and TB, wherein IFN activity in TB is not systemically present but is mostly confined to locally affected organs, whereas systemic IFN activity contributes to sarcoidosis. We propose that the measurement of serum IFNs and IFN activity is promising for discriminating between sarcoidosis/SU and TB/TBU, as summarized in Fig. 5. Implementing the tests in the current study in a diagnostic setting poses challenges. IFNα2 measurement using the Simoa platform is costly and requires pre-sample collection. Measuring the 10 ISGs in serum-stimulated THP-1 cell cultures is complex because of ongoing cell culture and serum stimulation requirements. These challenges underscore the need for novel and cost-effective strategies to routinely measure the serum IFN activity.

## Data availability statement

Data included in article/supp. material/referenced in article.

## CRediT authorship contribution statement

**Benjamin Schrijver:** Writing – review & editing, Writing – original draft, Visualization, Validation, Project administration, Investigation, Formal analysis, Data curation, Conceptualization. **Jens Göpfert:** Writing – review & editing, Formal analysis, Data curation. **Rina La Distia Nora:** Writing – review & editing, Conceptualization. **Ikhwanuliman Putera:** Writing – review & editing, Conceptualization. **Nicole M.A.N. Nagtzaam:** Writing – review & editing, Investigation, Formal analysis. **Marja A.W. Smits te Nijenhuis:** Writing – review & editing, Investigation, Formal analysis. **Angelique L.C.T. van Rijswijk:** Writing – review & editing, Investigation, Formal analysis. **Josianne C.E.M. ten Berge:** Writing – review & editing, Data curation, Conceptualization. **Jan A.M. van Laar:** Writing – review & editing, Data curation, Conceptualization. **P. Martin van Hagen:** Writing – review & editing, Supervision, Data curation, Conceptualization. **Willem A. Dik:** Writing – review & editing, Writing – original draft, Supervision, Conceptualization.

## Declaration of competing interest

The authors declare that they have no known competing financial interests or personal relationships that could have appeared to influence the work reported in this paper.

## Figures and Tables

**figs1:**
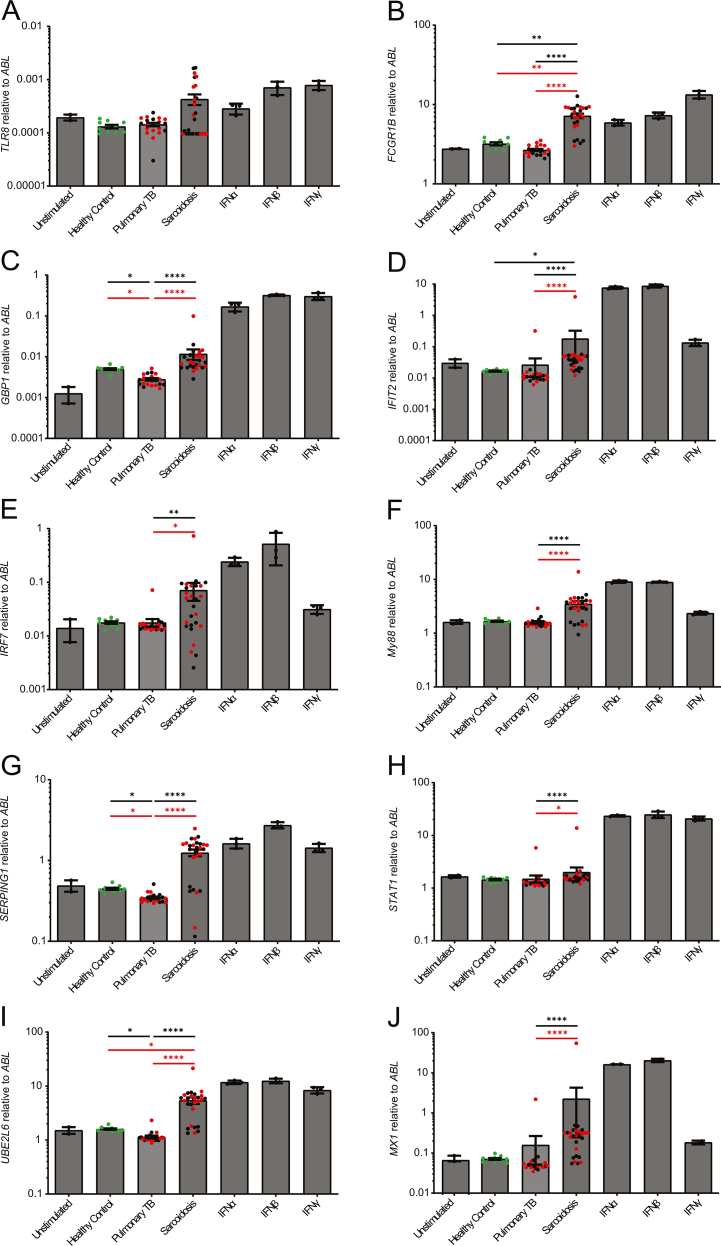


**figs2:**
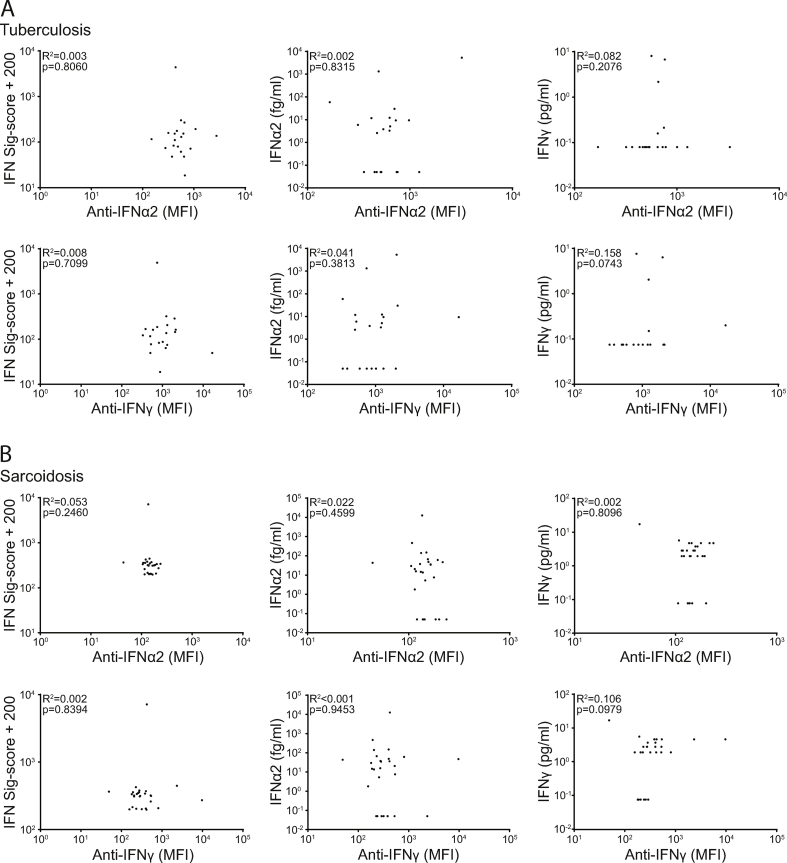


**figs3:**