# Supplementary material for: Increased serum interferon activity in sarcoidosis compared to that in tuberculosis: Implication for diagnosis?
Source: Heliyon. 2024 Aug 28;10(18):e37103. doi: 10.1016/j.heliyon.2024.e37103 (PMC11416298; doi:10.1016/j.heliyon.2024.e37103)
Supplement: Multimedia component 1 [file mmc1.docx]

­Supplementary Table 1. Patients with sarcoidosis

| **Patient number** | **Age** | **Gender** | **Lung involvement** | **Ocular involvement** | **Other Organ(s) Involvement** |
| --- | --- | --- | --- | --- | --- |
| 1 | 44 | Female | Yes | Yes | Skin |
| 2 | 33 | Female | Yes | Yes | Nervous system |
| 3 | 67 | Male | No | Yes | N/A |
| 4 | 50 | Male | Yes | No | Lymph nodes |
| 5 | 66 | Female | Yes | No | Lymph nodes |
| 6 | 45 | Male | Yes | Yes | N/A |
| 7 | 87 | Female | No | Yes | Lymph nodes |
| 8 | 51 | Male | Yes | Yes | N/A |
| 9 | 69 | Female | Yes | No | Skin, Parotid glands |
| 10 | 53 | Male | Yes | Yes | Muscles, Joints |
| 11 | 54 | Male | Yes | No | Skin, Joints |
| 12 | 67 | Female | Yes | No | Skin, Parotid glands |
| 13 | 40 | Male | Yes | Yes | Skin, Lymph nodes, ENT |
| 14 | 62 | Female | Yes | Yes | N/A |
| 15 | 21 | Female | No | Yes | N/A |
| 16 | 69 | Male | Yes | No | Skin, Stomach |
| 17 | 32 | Male | Yes | No | Nervous system |
| 18 | 35 | Female | Yes | Yes | N/A |
| 19 | 66 | Female | No | Yes | Lymph nodes |
| 20 | 50 | Female | Yes | No | Joints |
| 21 | 39 | Female | Yes | No | Liver |
| 22 | 79 | Female | Yes | No | N/A |
| 23 | 51 | Male | Yes | No | Kidney |
| 24 | 29 | Female | Yes | Yes | N/A |
| 25 | 54 | Female | Yes | Yes | Skin, Joints |
| 26 | 69 | Female | No | No | Skin |
| 27 | 45 | Male | No | Yes | Hearth |

ENT = ear, nose and throat
