# Supplementary material for: Increased serum interferon activity in sarcoidosis compared to that in tuberculosis: Implication for diagnosis?
Source: Heliyon. 2024 Aug 28;10(18):e37103. doi: 10.1016/j.heliyon.2024.e37103 (PMC11416298; doi:10.1016/j.heliyon.2024.e37103)
Supplement: Multimedia component 2 [file mmc2.docx]

­Supplementary Table 2. Primer-Probe Sets

| **Gene** |  |
| --- | --- |
| *ABL1* | TaqMan Gene Expression Assays (Hs99999002_mH), Life technologies |
| *TLR8* | TaqMan Gene Expression Assays (Hs00152972_m1), Life technologies |
| *FCGR1B* | TaqMan Gene Expression Assays (Hs00417598_m1), Life technologies |
| *GBP1* | TaqMan Gene Expression Assays (Hs00977005_m1), Life technologies |
| *IFIT2* | TaqMan Gene Expression Assays (Hs00533665_m1), Life technologies |
| *IRF7* | TaqMan Gene Expression Assays (Hs00185375_m1), Life technologies |
| *My88* | TaqMan Gene Expression Assays (Hs01573837_g1), Life technologies |
| *SERPING1* | TaqMan Gene Expression Assays (Hs00163781_m1), Life technologies |
| *STAT1* | TaqMan Gene Expression Assays (Hs01013996_m1), Life technologies |
| *UBE2L6* | TaqMan Gene Expression Assays (Hs01125548_m1), Life technologies |
| *MX1* | TaqMan Gene Expression Assays (Hs00895598_m1), Life technologies |

*ABL* = abelson murine leukemia viral oncogene homolog 1*, TLR8* = toll like receptor 8, *FCGR1B* = Fc fragment of IgG receptor Ib, *GBP1 =* guanylate binding protein 1, *IFIT2* = interferon induced protein with tetratricopeptide repeats 2, *IRF7* = interferon regulatory factor 7, *My88* = myeloid differentiation primary response 88, *SERPING1* = serpin family G member 1, *STAT1* = signal transducer and activator of transcription 1*, UBE2L6* = ubiquitin/ISG15-conjugating enzyme E2 L6, *MX1* = MX dynamin like GTPase 1
